# Evidence for Minimal Cardiogenic Potential of Stem Cell Antigen 1–Positive Cells in the Adult Mouse Heart

**DOI:** 10.1161/CIRCULATIONAHA.118.035273

**Published:** 2018-12-17

**Authors:** Lauren E. Neidig, Florian Weinberger, Nathan J. Palpant, John Mignone, Amy M. Martinson, Daniel W. Sorensen, Ingrid Bender, Natsumi Nemoto, Hans Reinecke, Lil Pabon, Jeffery D. Molkentin, Charles E. Murry, Jop H. van Berlo

**Affiliations:** 1 Department of Pathology (L.E.N., F.W., N.J.P., A.M.M., H.R., L.P., C.E.M.), University of Washington, Seattle.; 2 Institute for Stem Cell and Regenerative Medicine (L.E.N., F.W., N.J.P., J.M., A.M.M., H.R., L.P., C.E.M.), University of Washington, Seattle.; 3 Center for Cardiovascular Biology (L.E.N., F.W., N.J.P., J.M., A.M.M., H.R., L.P., C.E.M.), University of Washington, Seattle.; 4 Department of Medicine/Cardiology (J.M., C.E.M.), University of Washington, Seattle.; 5 Department of Comparative Medicine (L.E.N), University of Washington, Seattle.; 6 Department of Bioengineering (C.E.M.), University of Washington, Seattle.; 7 Stem Cell Institute and Lillehei Heart Institute, Department of Medicine, University of Minnesota, Minneapolis (D.W.S., I.B., N.N., J.H.v.B.).; 8 Department of Pediatrics, Howard Hughes Medical Institute, Cincinnati Children’s Hospital Medical Center, University of Cincinnati, OH (J.D.M.).; 9 F.W. is currently at the Department of Experimental Pharmacology and Toxicology, University Medical Center Hamburg-Eppendorf, Germany.; 10 J.M. is currently at the Cardiology Division, Swedish Medical Center, Seattle, WA.; 11 N.J.P. is currently at the Institute for Molecular Bioscience, University of Queensland, Brisbane, Australia.

**Keywords:** heart failure, myocardial infarction, progenitor cells, regeneration

**Editorial, see p 2940**

De novo cardiomyogenesis is limited to ≈1% per year in the adult mammalian heart.^1^ Whether newly formed cardiomyocytes are derived from division of pre-existing myocytes or from differentiation of resident cardiac progenitor cells is a topic of debate. Cardiac progenitor cells have been posited as a source of endogenous cardiomyocyte renewal and as cells that can be harvested, expanded in vitro, and delivered therapeutically after infarction. Stem cell antigen 1 (Sca-1), which was initially described as a surface marker of murine hematopoietic stem cells, has been reported to mark resident cardiac progenitor cells,^2^ and a previous study reported frequent contribution of Sca-1–expressing cells to cardiomyocytes.^3^ However, the transgenic approach used in this study resulted in more widespread expression than just Sca-1–expressing cells, complicating interpretation of the results.^4^ Because more recent fate mapping studies questioned the existence of resident cardiac progenitor cells, we aimed to test the hypothesis that endogenous Sca-1–expressing cells are progenitors for cardiomyocytes in vivo under physiological and pathophysiological conditions.

We generated a tamoxifen-inducible genetic lineage-tracing Sca-1 mER-Cre-mER knock-in mouse model (Sca-1^mCm/+^)^5^ and cross-bred to a Rosa26 tdTomato reporter line (Sca-1^mCm^R26^tdTomato^) to assess the fate of cellular descendants of Sca-1–expressing cells in vivo. Adult Sca-1^mCm^R26^tdTomato^ animals were treated with tamoxifen daily for 7 days, and hearts were harvested 7 days after the last tamoxifen injection to assess recombination. Recombination in the heart averaged 33% in Sca-1^+^/CD31^+^ populations and 13% in the Sca-1^+^/CD31^–^ population (Figure [A] and [B]). No tdTomato^+^ cells were identified by flow cytometry in Sca-1^mCm^R26^dTomato^ mice that did not receive tamoxifen. Histology (Figure [C]) demonstrated a mostly endothelial expression pattern in Sca-1^mCm^R26^dTomato^ hearts that colocalized with CD31 expression. Additionally, there was overlap between Sca-1 and tdTomato staining as expected, as well as some NG2-expressing perivascular cells that overlapped with tdTomato. In tissue sections, TdTomato was not identified in cardiomyocytes. Additionally, tamoxifen-dependent tdTomato expression in tissues known to express Sca-1 (kidney, lung, liver, ileum) confirmed the presence of tdTomato^+^ cells, predominately in an endothelial pattern; this was absent in tissue from animals that received no tamoxifen (data not shown).

**Figure.**
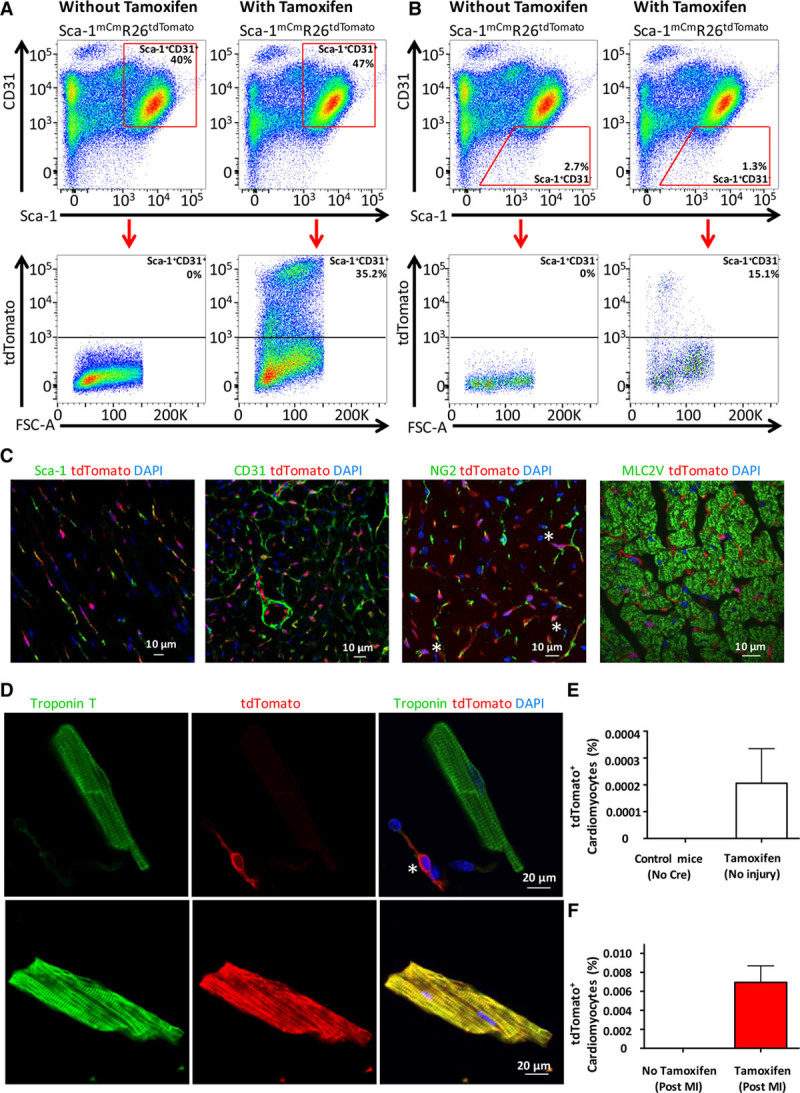
**Lineage tracing of Sca-1 expressing cells in the mouse heart. A**, Stem cell antigen 1 (Sca-1)^+^CD31^+^ cells isolated from Sca-1^mCm^R26^tdTomato^ hearts demonstrated successful recombination after tamoxifen induction (right) without leakiness in the absence of tamoxifen (left). **B**, Sca-1^+^CD31^−^ cells isolated from Sca-1^mCm^R26^tdTomato^ hearts demonstrated successful recombination after tamoxifen induction (right) without leakiness in the absence of tamoxifen (left). **C**, TdTomato expression demonstrated a vascular staining pattern that overlapped with Sca-1 and CD31 staining. tdTomato expression sometimes overlapped with the perivascular marker NG2, as illustrated by the asterisks. tdTomato expression did not overlap with the cardiomyocyte marker MLC2V. Cell nuclei were stained with 4’,6-diamidino-2-phenylindole (DAPI). **D**, Enzymatic digestion allowed for unambiguous differentiation between cardiomyocytes and nonmyocytes. The asterisk indicates a tdTomato^+^ nonmyocyte in the top panel, whereas the cardiac troponin T (cTnT)^+^ myocyte in the same image is tdTomato-negative. The bottom panel is an example of a rare tdTomato^+^ cardiomyocyte. **E**, Quantification of tdTomato^+^ cardiomyocytes 7 days after tamoxifen from live cardiomyocytes. **F**, After myocardial infarction (MI), a total of 19 tdTomato+ cardiomyocytes were detected in 7 animals, accounting for a derived average percentage of 0.007% Sca-1^+^–derived cardiomyocytes (error bar = SEM). No tdTomato+ cardiomyocytes were observed in the absence of tamoxifen treatment. FSC-A indicates forward-scatter area.

Next, we set out to test whether new cardiomyocytes were derived from descendants of Sca-1–expressing cells after myocardial infarction (MI). Adult Sca-1^mCm^R26^dTomato^ mice were treated with tamoxifen daily for 7 days, followed by a 7-day chase period. The mice then underwent MI or were allowed to age normally. Mice were euthanized 6 months later for examination of the heart. We enzymatically dissociated cardiomyocytes and quantified either fraction of tdTomato^+^ cardiomyocytes from live cells (uninjured), or after immunostaining for cardiac troponin T and tdTomato (post-MI). This allowed unambiguous distinction of cardiomyocytes and nonmyocytes (Figure [D]). We first assessed the numbers of tdTomato^+^ cardiomyocytes at baseline before injury and detected 6 tdTomato^+^ cardiomyocytes from 5 hearts (0.0002%; Figure [E]). We then assessed labeling of cardiomyocytes after MI and detected a total of only 19 tdTomato^+^ cardiomyocytes from 7 hearts (Figure [F]). This accounts for 0.007% tdTomato^+^ cardiomyocytes after infarction on average. Importantly, not a single cardiomyocyte expressed tdTomato in animals that were not exposed to tamoxifen, demonstrating that this is not a leaky tracing system.

The results of this study do not support the hypothesis that Sca-1–expressing cells have the ability to significantly contribute new cardiomyocytes to the heart after MI in the mouse. Despite using the best available techniques for lineage tracing, including knocking a tamoxifen-inducible Cre recombinase into the endogenous Sca-1 gene, using a sensitive Cre-reporter strain, and enzymatically dispersing cardiomyocytes for single-cell screening, we were unable to detect significant contributions of the Sca-1 lineage to cardiomyocytes.

Our study contrasts the study by Uchida et al,^3^ which showed a significant contribution of Sca-1^+^ cells to cardiomyocytes. The main difference between these 2 studies relates to the lineage-tracing approach (ie, a transgenic promoter fragment [Uchida et al] versus the endogenous Sca-1 locus dependent Cre expression [this study]). The transgenic system used by Uchida et al has been reported to generate a large number of false-positives, meaning the promoter fragment is active in cells that normally do not express Sca-1.^4^ Therefore, we believe the transgenic promoter fragment may have overestimated the true cardiogenic potential of endogenous Sca-1–expressing cells in this model. In our model, the recombination rates in the heart were reasonable, but still only resulted in 0.007% of cardiomyocytes to become labeled after MI. Thus, although this number might be an underestimation, it is 2 orders of magnitude too low to explain a 1% per year cardiomyocyte turnover rate. When coupled with data demonstrating that most cardiomyocyte renewal can be accounted for by division of pre-existing cardiomyocytes, our findings reduce the likelihood that there is a cardiac progenitor cell population in mammalian species.

All animal procedures complied with and were approved by the Institutional Animal Care and Use Committee.

## Acknowledgments

We thank Dr Kyohei Oyamaya for expert training in cardiomyocyte isolation, and Dr Dale Hailey in the Mike and Lynn Garvey Imaging Core within the Institute for Stem Cell and Regenerative Medicine for assistance with fluorescent imaging.

## Sources of Funding

This study was supported in part by a grant from the Fondation Leducq Transatlantic Network of Excellence and National Institutes of Health grants P01HL094374, R01HL128362, GM081619 (to C.E.M.), R00HL112852, and R01HL130072 (to J.H.v.B.), and a research scholarship from the German Research Foundation (to F.W.). D.W.S. is supported by National Institutes of Health grant T32GM113846. This research also was supported by the Cell Analysis Facility Flow Cytometry and Imaging Core in the Department of Immunology at the University of Washington.

## Disclosures

Dr Murry is a scientific founder and equity holder in Cytocardia. The other authors report no conflicts.
